# Morphological, behavioral and cellular analyses revealed different phenotypes in Wolfram syndrome *wfs1a* and *wfs1b* zebrafish mutant lines

**DOI:** 10.1093/hmg/ddac065

**Published:** 2022-04-22

**Authors:** Lucie Crouzier, Elodie M Richard, Camille Diez, Hala Alzaeem, Morgane Denus, Nicolas Cubedo, Thomas Delaunay, Emily Glendenning, Sarah Baxendale, Jean-Charles Liévens, Tanya T Whitfield, Tangui Maurice, Benjamin Delprat

**Affiliations:** MMDN, Université Montpellier, EPHE, INSERM, Montpellier, France; MMDN, Université Montpellier, EPHE, INSERM, Montpellier, France; MMDN, Université Montpellier, EPHE, INSERM, Montpellier, France; MMDN, Université Montpellier, EPHE, INSERM, Montpellier, France; MMDN, Université Montpellier, EPHE, INSERM, Montpellier, France; MMDN, Université Montpellier, EPHE, INSERM, Montpellier, France; IES, Université Montpellier, CNRS, Montpellier, France; Development, Regeneration and Neurophysiology, School of Biosciences, University of Sheffield, Sheffield S10 2TN, UK; Development, Regeneration and Neurophysiology, School of Biosciences, University of Sheffield, Sheffield S10 2TN, UK; MMDN, Université Montpellier, EPHE, INSERM, Montpellier, France; Development, Regeneration and Neurophysiology, School of Biosciences, University of Sheffield, Sheffield S10 2TN, UK; MMDN, Université Montpellier, EPHE, INSERM, Montpellier, France; MMDN, Université Montpellier, EPHE, INSERM, Montpellier, France

## Abstract

Wolfram syndrome (WS) is a rare genetic disease characterized by diabetes, optic atrophy and deafness. Patients die at 35 years of age, mainly from respiratory failure or dysphagia. Unfortunately, there is no treatment to block the progression of symptoms and there is an urgent need for adequate research models. Here, we report on the phenotypical characterization of two loss-of-function zebrafish mutant lines: *wfs1a*^*C825X*^ and *wfs1b*^*W493X*^. We observed that *wfs1a* deficiency altered the size of the ear and the retina of the fish. We also documented a decrease in the expression level of unfolded protein response (UPR) genes in basal condition and in stress condition, i.e. after tunicamycin treatment. Interestingly, both mutants lead to a decrease in their visual function measured behaviorally. These deficits were associated with a decrease in the expression level of UPR genes in basal and stress conditions. Interestingly, basal, ATP-linked and maximal mitochondrial respirations were transiently decreased in the *wfs1b* mutant. Taken together, these zebrafish lines highlight the critical role of *wfs1a* and *wfs1b* in UPR, mitochondrial function and visual physiology. These models will be useful tools to better understand the cellular function of Wfs1 and to develop novel therapeutic approaches for WS.

## Introduction

Wolfram syndrome (WS) is an autosomal recessive genetic disorder characterized by diabetes mellitus, diabetes insipidus, optic nerve atrophy, hearing loss and neurodegeneration (1). This disease is progressive and severe, leading to the premature death of affected individuals around 35 years of age, with severe neurological disabilities, including bulbar dysfunction and organic brain syndrome (2). More than 200 variants in the *WFS1* gene, which encodes the endoplasmic reticulum (ER)-resident glycoprotein Wolframin (WFS1) (3,4), have been associated so far with the disease (5).

The unfolded protein response (UPR) is an adaptive response to ER stress, with activation of RNA-activated protein kinase-like endoplasmic reticulum kinase (PERK), activating transcription factor 6 (ATF6) and/or inositol-requiring kinase 1 (IRE1), preventing the accumulation of stress-induced unfolded proteins and improving ER homeostasis (6). Under ER stress, ATF6 dissociates from WFS1 and is cleaved and released from the ER to regulate stress signaling targets in the nucleus. As ER homeostasis is restored, WFS1 expression is induced by ER stress, which results in the degradation of ATF6 (7). In WS, WFS1 is not functional. ATF6 is no longer degraded via WFS1 and is therefore overactivated, regardless of emergency stress conditions, leading to the death of pancreatic β cells (8).

WFS1 also plays a critical role in calcium homeostasis regulation. Under physiological conditions, WFS1 interacts with the Ca^2+^ sensor NCS1 (9) and prevents its degradation, both proteins forming a complex with the inositol-1,4,5 triphosphate receptor (IP3R) to activate ER-Ca^2+^ transfer to mitochondria. When the tripartite WFS1/NCS1/IP3R complex and VDAC1 are functional, Ca^2+^ can properly transfer from the ER to the mitochondria and activate the tricarboxylic acid cycle and mitochondrial oxidative respiration. We have previously shown that under WS pathophysiological conditions, the WFS1/NCS1/IP3R complex loses its effectiveness, leading to the degradation of NCS1 and a decrease in ER-Ca^2+^ transfer to mitochondria, triggering *in fine* cell death (9,10).

Currently, no treatment for WS is available and patients have only access to medical devices that would improve their quality of life. The urgency to find a cure is obvious. Cellular (11–13) and murine models (14–17) have been developed to reproduce various deficits observable in humans and, therefore, to understand the pathological mechanisms and alleviate the alterations with active molecules.

In recent years, the zebrafish (*Danio rerio*) has become an alternative research model. It offers a unique combination of different characteristics, including short generation time, transparency, small size and very efficient breeding methods, and its genome is very close to that of humans. These properties make it ideal for studying genes associated with human diseases (18–20), particularly neurodegenerative diseases and neurological disorders (21–24). They also confer great advantages to the zebrafish model compared with other models for finding new treatments, most particularly for testing a very large number of potential therapeutic molecules quickly and at lower cost as well as directly testing on a whole living organism with all its complexity (25,26).

We here characterized two zebrafish lines that carry stop codon mutations in orthologs of the *WFS1* gene, which are responsible for WS. Their precise phenotyping was necessary to better understand the physiopathology and to realize their usefulness as a screening model for the validation of novel therapeutic strategies, such as repurposed drugs, ER stress chaperones or potential mitoprotectants. As the zebrafish genome is duplicated, the impact of *wfs1* mutations in both copies of the gene, *wfs1a* and *wfs1b*, could be addressed using two mutant lines: *wfs1a^C825X^* and *wfs1b^W493X^*. We investigated, at the behavioral level, their roles in sensory functions such as vision or hearing and locomotion and, at the cellular level, their impact on metabolic markers like ER stress factors, glycemia and mitochondrial function.

## Results

### Morphology and grossly observable phenotypes of *wfs1a* and *wfs1b* mutant zebrafish lines

**Figure 1:**
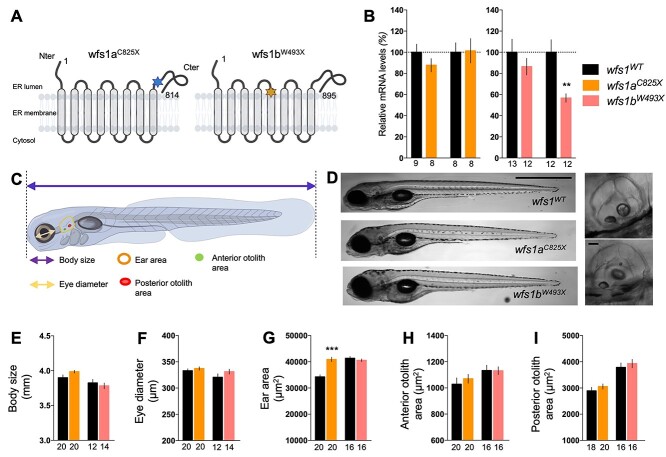
Characterization of *wfs1* mutant zebrafish lines. (**A**) Schematic molecular structure of Wfs1a and Wfs1b proteins with mutation. The stars represent the punctual mutations, blue for Wfs1a mutation and orange for Wfs1b. (**B**) Relative *wfs1a* and *wfs1b* mRNA levels assessed by qPCR in *wfs1a^C825X^* and *wfs1b^W493X^* zebrafish larvae, normalized against *zef1α* reference gene. (**C**) Schematic representation of the different measurements of the larva. (**D**) Representative images of wild-type and homozygous mutant *wfs1* larvae. Images to the right show the ear in the wild-type (upper image) and *wfs1a^C825X^* mutant (the lower image). (**E**) Measurement of body size, (**F**) eye diameter, (**G**) ear area in lateral view, (**H**) anterior and (**I**) posterior otoliths area. Scale bars, 200 μm in (D), 10 μm in the insets. Error bars represent mean ± SEM and the number of fish is indicated below the columns. ^**^P < 0.01, ^***^P < 0.0001; unpaired *t*-test.

Various mutant lines for *wfs1a* and *wfs1b* were generated through ENU mutagenesis (27). For each gene, the line carries a premature stop codon, leading, potentially, to a non-functional protein. We selected the *wfs1a^C825X^* (sa11465) and *wfs1b^W493X^* (sa16422) lines (Fig. 1A) in which point mutations lead to a premature stop codon and potentially to a non-functional protein. These mutations abolished restriction sites for *Mwo*I and *Bsr*I, respectively, allowing each genotype to be validated by PCR followed by enzymatic digestion (Supplementary Material, Fig. S1A). Sanger sequencing confirmed the non-sense mutations (Supplementary Material, Fig. S1B).

In order to determine if the mutations induced mRNA decay, relative *wfs1a* and *wfs1b* mRNA levels were measured by quantitative real-time PCR (qPCR) in both mutant lines (Fig. 1B). Surprisingly, neither *wfs1a* nor *wfs1b* gene expression was altered in *wfs1a^C825X^* mutants, whereas both genes were downregulated in *wfs1b^W493X^* fish, suggesting a genetic interaction of *wfs1b* on the *wfs1a* gene. The protein expression level of Wfs1 could not be assessed as no effective antibodies specifically labeling the zebrafish protein are presently available. However, owing to the premature stop codons, we predicted that both proteins, when expressed, are not functional.

In order to determine if mutations in *wfs1* have an impact on zebrafish larval morphology, different parameters, detailed in Figure 1C, were measured at 5 dpf using a binocular magnifier (Fig. 1D) and were analyzed using Fiji software (Fig. 1E–I). Overall, the mutations did not impact the observable gross morphology of the larva, with the exception of the enlarged ear area for *wfs1a^C825X^* compared with controls (Fig. 1D and G).

### *wfs1a* and *wfs1b* are expressed in distinct but overlapping domains during zebrafish development

**Figure 2:**
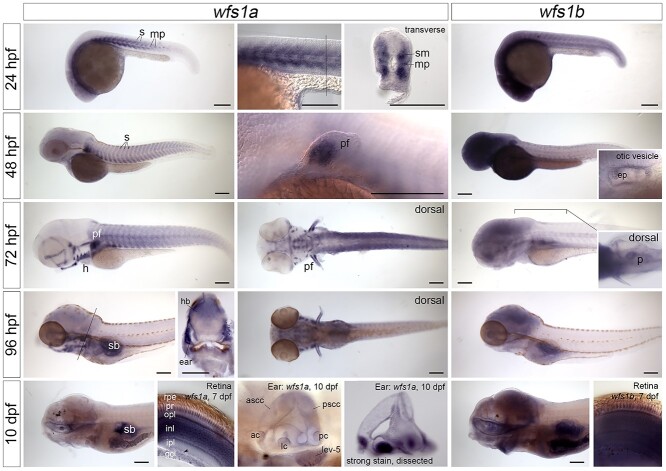
Expression of *wfs1a* and *wfs1b* mRNA in the zebrafish embryo, larva and juvenile. *In situ* hybridizations showing the expression patterns of *wfs1a* (left-hand and middle columns) and *wfs1b* (right-hand column) between 24 hpf and 10 dpf. All images are lateral views with anterior to the left, except for the inset showing expression of *wfs1b* in the pancreas (dorsal view, anterior to the left) and the sections through the retina. The position of the transverse hand-cut sections through the trunk at 24 and 96 hpf (dorsal to the top) is marked by a thin line in the preceding image. The eyes have been removed from the 10 dpf embryos, to show expression in the mouth and brain. The hand-cut thick sections through the retina, and the second image of the ear at 10 dpf, were taken from samples stained using an alternative protocol that resulted in stronger staining intensity (see Materials and Methods). Expression of *wfs1a* in the retina at 7 dpf is strong in the photoreceptors, outer plexiform and inner nuclear layers but is weaker or missing from the inner plexiform and ganglion cell layers (GCLs) and is very weak or absent from displaced amacrine cells (arrowhead). Expression of *wfs1b* in the retina at 7 dpf appears ubiquitous. Expression in the inner ear appears ubiquitous for both genes and may include some trapping. All images are bright-field images apart from the images of the trunk at 24 hpf, pectoral fin at 48 hpf and retinae at 7 dpf, which were taken with DIC optics. Abbreviations: ac, anterior crista; ascc, anterior semicircular canal; ep, epithelial projections in the otic vesicle; gcl, ganglion cell layer; h, heart; hb, hindbrain; inl, inner nuclear layer; ipl, inner plexiform layer; lc, lateral crista; lev-5, levator arcus branchialis 5; mp, muscle pioneer cells; opl, outer plexiform layer; p, pancreas; pc, posterior crista; pf, pectoral fin; pr, photoreceptors; pscc, posterior semicircular canal; rpe, retinal pigmented epithelium; s, somites; sb, swimbladder; sm, slow-twitch muscle fibers. Scale bars, 200 μm in all images.

To characterize the mRNA expression patterns of *wfs1a* and *wfs1b* during development, two antisense RNA probes for each gene were generated and were used for *in situ* hybridization on zebrafish embryos, larvae and juveniles at different developmental stages (Fig. 2). At early stages [24 h post-fertilization (hpf)], expression patterns of the two paralogs were quite distinct, with specific expression of *wfs1a* in developing skeletal muscle, whereas expression of *wfs1b* was more ubiquitous. Transverse sections through the trunk at 24 hpf showed *wfs1a* expression in the developing somites, most strongly in the slow-twitch muscle fibers and muscle pioneer cells. Strong expression in muscles of the pectoral fin was evident from 48 hpf. Expression in the craniofacial skeletal muscles, including those articulating the eye and the jaw, was present from 72 hpf. By 96 hpf, the expression of *wfs1a* in trunk somitic muscle was reduced, but a strong expression in craniofacial skeletal muscle was maintained. Weak expression of *wfs1a* was also present in the heart (most likely myocardium) from 72 hpf, and in the mouth, swimbladder, gut and brain from 96 hpf. Expression of *wfs1b* was ubiquitous at early stages, with strong expression in the central nervous system (brain and retina), persisting in the brain up to 10 days post-fertilization (10 dpf). From 48 hpf, expression of *wfs1b* was also present in the exocrine pancreas, which was visible in a dorsal view as a characteristic comma-shaped organ on the right-hand side of the body (see Fig. 2, inset). At later stages, there were several regions of overlap in the expression patterns of *wfs1a* and *wfs1b*. Both genes were expressed in the retina at 7 dpf, with *wfs1a* showing different levels of expression in the different retinal cell layers. Both genes were also weakly expressed in otic epithelium, including in the epithelial projections that lead to the formation of the semicircular canals at 48 hpf (see inset). Using an adapted staining protocol (see Materials and Methods), we detected expression of both zebrafish genes throughout the brain, but not the spinal cord, at 7 and 10 dpf. In the brain, expression marked the cell bodies but not the areas of neuropil (Supplementary Material, Fig. S2). Other sites of co-expression included the somites, pectoral fin and lens of the eye at 24–48 hpf and the brain, swimbladder, gut and gills at 7–10 dpf (Fig. 2, Supplementary Material, Fig. S2).

### The *wfs1* mutations impacted visual and locomotor functions of zebrafish larvae

**Figure 3:**
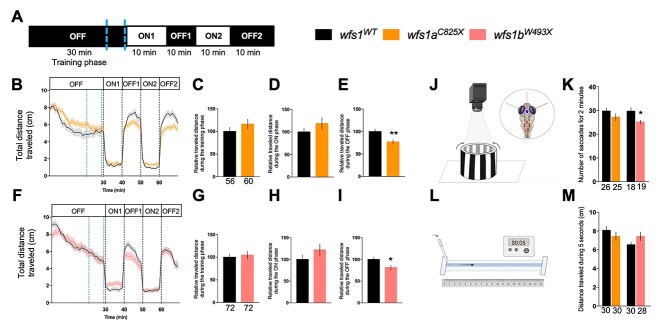
Behavioral analyses of 5 dpf *wfs1* mutant larvae. (**A**) Light protocol: the activity is measured for 70 min, with 30 min of training in the dark (OFF), then two cycles of light/dark (ON/OFF) of 10 min each. Analysis of the distance traveled by (**B**) the *wfs1a^C825X^* and (**F**) *wfs1b^W493X^* larvae at 5 dpf during the light/dark cycle in the VMR test. Relative distance traveled was measured during some experiment phases as (**C**, **G**) the training phase [blue-dotted lines in (B, F) between 21 and 29 min]; (**D**, **H**) the ON phase (the averaged ON1 and ON2 phases); (**E**, **I**) the OFF phase (the averaged OFF1 and OFF2 phases). (**J**) Illustration of the OKR test. Four larvae are immobilized in petri dish and placed in an arena with moving black and white gratings. (**K**) The number of saccades performed in 2 min. (**L**) Illustration of the touch escape test. The larva’s tail is touched with a tip and (**M**) the traveled distance of the larva in the rail is measured for 5 s, repeated for three times per larva and averaged. Relative distances were expressed as % of associated controls. Error bars represent ± SEM calculated from three replicas. The number of animals is indicated below the columns. ^*^P < 0.05; ^**^P < 0.01; unpaired *t*-test.

The locomotor response of larvae after visual stimulation was analyzed using the visual motor response (VMR) assay. At 5 dpf, larvae were placed individually in the wells of a 96-well plate and their individual activity was measured according to the described protocol. A training phase of 30 min in the dark (OFF, 0% light) allowed the larva to acclimate to its new environment, followed by two sequences of light (ON, 100% light, 10 min) and dark periods (OFF, 0% light, 10 min) (Fig. 3A). For both lines, the traveled distance profiles are shown in Figure 3B and F; and the quantification is shown in Figure 3C and G for the training phases; in Figure 3D and H, for the ON periods; and in Figure 3E and I, for the OFF periods. In the *wfs1a^C825X^* (Fig. 3B–E) and *wfs1b^W493X^* mutant lines (Fig. 3F–I), the relative distance traveled during the training (Fig. 3C and G) and ON (Fig. 3D and H) phases were not altered. However, the distance traveled during the OFF period (Fig. 3E and I) phase was significantly decreased in both *wfs1a* and *wfs1b* mutant larvae.

The visual acuity of the two *wfs1* mutant zebrafish lines was measured using the optokinetic response (OKR) assay. The eye movement of the larva was induced by the rotation of black-and-white stripes retroprojected on a fixed cylinder (Fig. 3J). The number of saccades in 2 min reflected the visual acuity of the immobilized larva. The *wfs1a* mutant larvae did not show alteration in the number of saccades, but *wfs1b* mutants showed a significant decrease (Fig. 3K), suggesting a specific visual deficit in this line.

A reflex motor response was also measured using the touch-evoked escape behavior (Fig. 3L). After manual stimulation, none of the mutant line exhibited an alteration of the distance traveled, suggesting an absence of motor alteration (Fig. 3M).

**Figure 4:**
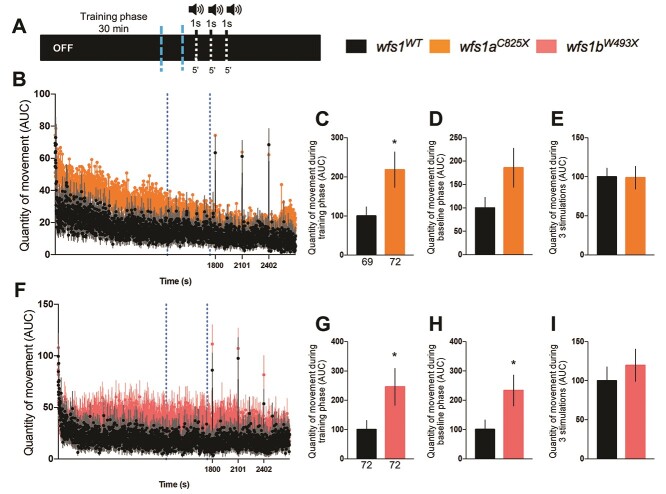
Analysis of the quantity of movement of 5 dpf *wfs1* mutant larvae during the noise cycle in the ASR test. (**A**) Sound protocol: the activity is measured for 45 min in a total dark (OFF) condition, with a training phase for 30 min silently, then three cycles of white sounds (90 dB) of 1 s each and 5 min interspersed. Quantity of movement per second for (**B**) the *wfs1a^C825X^* and (**F**) *wfs1b^W493X^* larvae according to the sound protocol. Relative quantity of movement during (**C**, **G**) the training phase [blue-dotted lines in (B, F) between 21 and 29 min]; (**D**, **H**) the baseline phase, period of 2 min before each sound (the averaged three baseline phases); (**E**, **I**) the three stimulations (the averaged sounds phases). Activity was expressed as % of associated controls. Error bars represent ± SEM calculated from three replicas. The number of animals is indicated below the columns. ^*^P < 0.05; unpaired *t*-test*.*

The acoustic startle response (ASR) of the larvae, induced by a repetitive 1 s duration noise (Fig. 4A), was assessed by measuring the quantity of movement in both *wfs1* mutant larvae (Fig. 4B and F). The quantity of movement during the training and baseline phases was increased in both *wfs1* mutant lines compared with controls (Fig. 4C, D, G and H). The *wfs1* mutations had, however, no impact on the quantity of movement after stimulation in either *wfs1* mutant line (Fig. 4E and I).

### The *wfs1a*^*C825X*^ mutation disrupted eye development of zebrafish larvae

**Figure 5:**
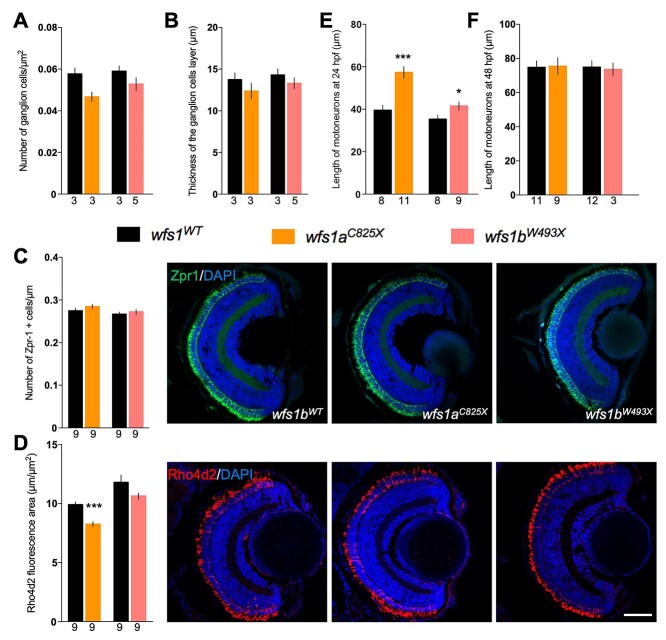
Molecular characterization of the retina and measure of the motor neuron length for both *wfs1* mutant zebrafish lines. Retina measurements in *wfs1* mutants with (**A**) quantification of the number of ganglion cells, (**B**) the thickness of the associated layer and (**C**) photoreceptors cells as red and green cones labeled with Zpr-1 antibody and (**D**) rods with anti-Rho4d2 antibody. Quantification of the tail motor neuron length of 24 hpf (**E**) and 48 hpf (**F**) zebrafish. For each fish, five to six neurons were measured, and the average length was calculated. Scale bar, 50 μm. The number of animals is indicated below the columns. ^*^P < 0.05; ^***^P < 0.001; unpaired *t*-test.

Immunohistochemical analysis of the developing eye was performed for both *wfs1a* and *wfs1b* lines (Fig. 5, Supplementary Material, Fig. S3). At 5 dpf, we observed a trend to a decrease in the number of retinal ganglion cells in *wfs1a^C825X^* compared with *wfs1a^WT^* controls (Fig. 5A, Supplementary Material, Fig. S3) without alteration of the thickness of the cell layer (Fig. 5B, Supplementary Material, Fig. S3). The number of red and green cones, labeled with Zpr-1 antibody, remained the same between mutant and control larvae (Fig. 5C). In contrast, the number of rods, labeled with Rho4d2 antibody, was significantly decreased (Fig. 5D).

### The *wfs1* mutations disrupted motor neuron length of 24 hpf zebrafish larvae

At 24 and 48 hpf, the immunohistochemical analysis of the developing motor neurons was performed for both *wfs1a* and *wfs1b* lines using SV2 antibodies (Fig. 5E and F). At 24 hpf, we observed a statistically significant increase in the length of *wfs1a^C825X^* and *wfs1b^W493X^* larvae motor neurons compared with their respective controls (Fig. 5E). This difference is no longer present at 48 hpf (Fig. 5F).

### The *wfs1* mutations affected the expression of ER stress genes

**Figure 6:**
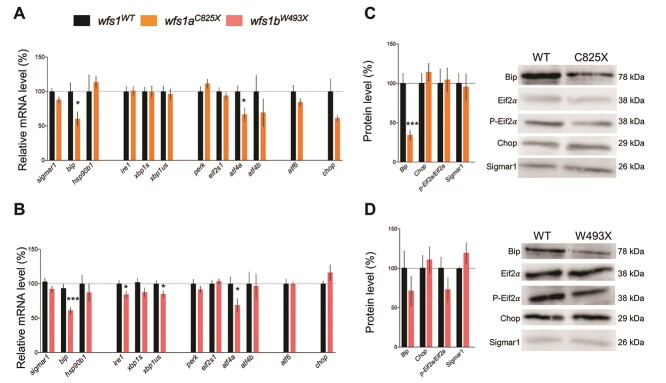
Relative mRNA and protein expression levels of larvae zebrafish ER stress factors in physiological condition at 5 dpf. mRNA levels were analyzed by qPCR and protein contents by western blot in (**A**, **C**) *wfs1a^C825X^* and (**B**, **D**) *wfs1b^W493X^*. *zeif2α* and Stain-free™ were used as controls in qPCR and western blot analysis, respectively. The expression levels of mRNA and protein are represented as the fold change from the control group. Data are expressed in mean ± SEM, *n* = 5–9 in each group. ^*^P < 0.05, ^***^P < 0.001; unpaired *t*-test.

The expression levels of genes involved in the regulation cascade of ER stress were analyzed by qPCR in both mutant zebrafish lines (Fig. 6A and B). Expression levels of the UPR pathway inducer, *hsp90b1*, were not modified in mutant larvae in all zebrafish lines, whereas *bip* mRNA levels were decreased in *wfs1a^C825X^* and *wfs1b^W493X^* compared with the controls (Fig. 6A and B). *Sigmar1* mRNA level was unchanged in either mutant line. Among the primary effectors of the UPR pathway, the expression of *ire1* was decreased only in *wfs1b^W493X^* compared with WT controls, while *perk*, *atf6* and *chop* were unchanged in both mutant lines. Among the secondary effectors, *xbp1us* was decreased in *wfs1b^W493X^* mutants compared with the controls (Fig. 6B) and *atf4α* was decreased in both *wfs1a^C825X^* and *wfs1b^W493X^*, while *xbp1s*, *eif2s1* and *atf4β* were unchanged in both lines.

Western blot analyses showed that the Bip protein level was significantly decreased in *wfs1a^C825X^* line and non-significantly altered in *wfs1b^W493X^* compared with controls, while there was no impact of *wfs1* mutations on expression levels of p-Eif2α/Eif2α and Sigmar1 (Fig. 6C and D).

### The *wfs1* mutations impact the tunicamycin-induced ER stress response

**Figure 7:**
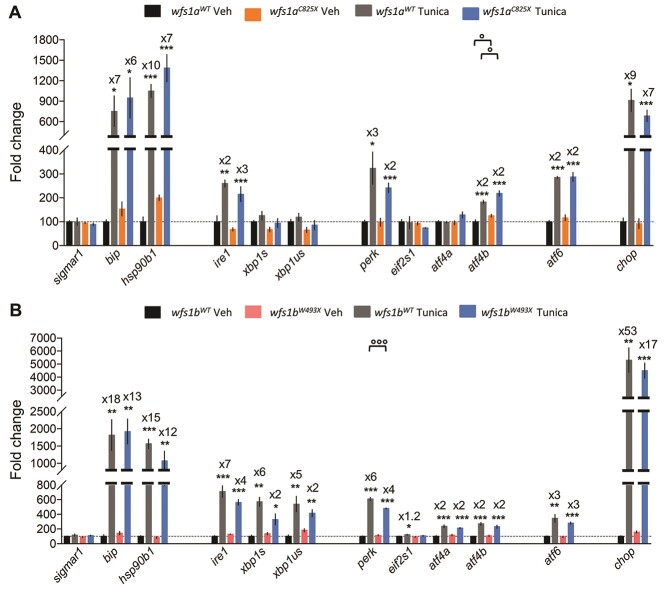
Relative gene expression levels of ER stress factors in zebrafish larvae at 5 dpf exposed to tunicamycin during 24 h. Expression analysis of the selected genes using cDNA prepared from *wfs1a^C825X^* (**A**) and *wfs1b^W493X^* (**B**) zebrafish larvae. The relative expression levels of mRNA are represented as the fold change from the control vehicle group. Data are expressed in mean ± SEM, *n* = 5 in each group. ^*^P < 0.05, ^**^P < 0.01, ^***^P < 0.001 versus DMSO condition*, °P* < 0.05, *°°°P* < 0.001 versus WT condition; two-way ANOVA, unpaired *t*-test.

Larvae were treated with 2 μg/ml of tunicamycin for 24 h to analyze the response of *wfs1* mutant lines to ER stress (Fig. 7). The mRNA levels of *bip*, *hsp90b1*, *ire1*, *perk*, *atf4b*, *atf6* and *chop* (Fig. 7A and B) were drastically increased in both mutant and control lines after the tunicamycin treatment, with the exception of *eif2s1* and *sigmar1* (Fig. 7A and B), which were unchanged with the treatment regardless of the genotype.

**Figure 8:**
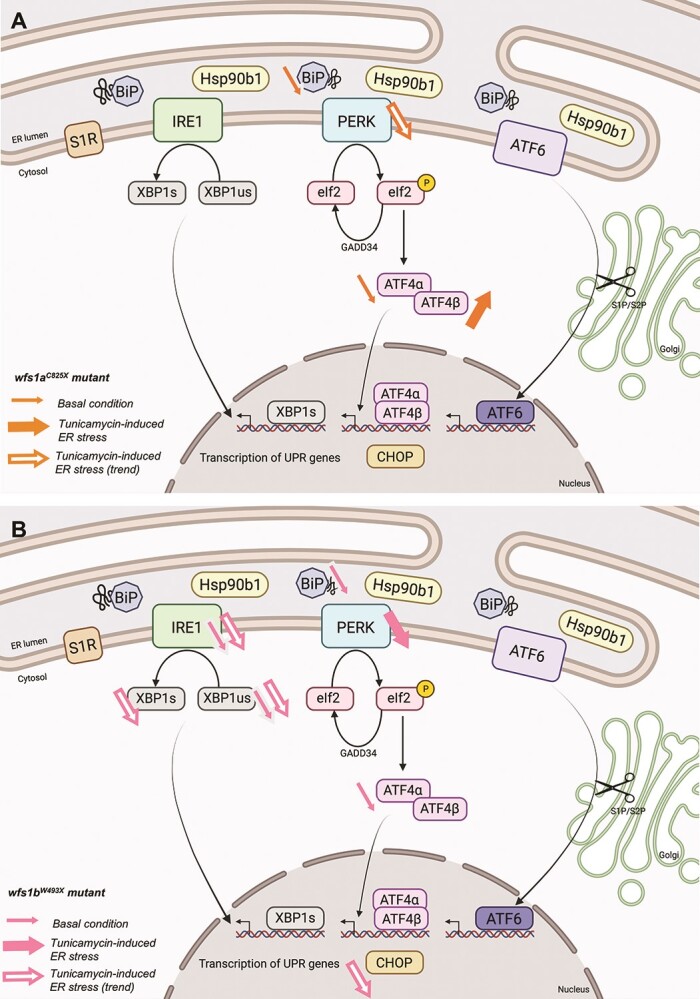
Schematic summary of ER stress pathways alterations observed in basal condition or after tunicamycin treatment in *wfs1a^C825X^* (**A**) *and wfs1b^W493X^* (**B**) zebrafish line.

Interestingly, the genotype has an impact on the ER stress response to tunicamycin treatment. In the *wfs1a* mutant line, primary sensors *ire1*, *perk* and *atf6* were increased but not the secondary effectors (Fig. 7A). In the *wfs1b* mutant line, *perk* was less induced by the tunicamycin treatment, but secondary effectors of both the *ire1* and *perk* pathways were significantly induced by tunicamycin (Fig. 7B). Taking into account the percentages of induction provoked by tunicamycin for each effector in the mutant and control lines, specified in Figure 7A and B, we summarized the observations in synthetic schemes presented for the *wfs1a^C825X^* and *wfs1b^W493X^* mutant lines. It appeared that UPR is marginally affected in the *wfs1a^C825X^* mutant line (Fig. 8A), while both *ire1*- and *perk*-dependent pathways are altered in the *wfs1b^W493X^* mutant line (Fig. 8B). These observations suggested that *wfs1* participated in ER stress regulation and that the *wfs1b* mutant zebrafish line is an adequate model to analyze the protein involvement in UPR (Fig. 8).

### Only *wfs1b* mutants showed a transient mitochondrial defect

**Figure 9:**
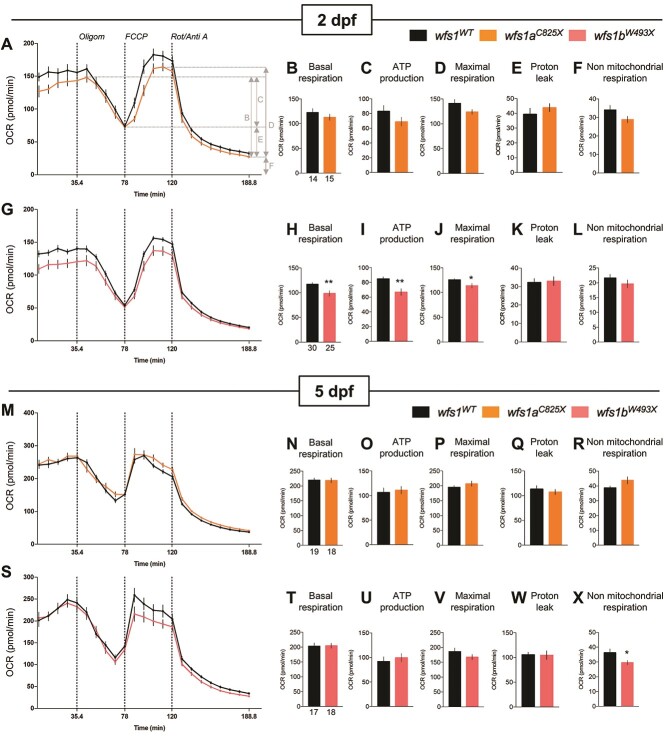
Analysis of mitochondrial respiration in zebrafish larvae at 2 and 5 dpf with Seahorse XF mito stress test. OCR profiles of (**A**–**L**) *wfs1a^C825X^* at 2 dpf (A–F) and 5 dpf (G–L); and OCR profiles of (**M**–**X**) *wfs1b^W493X^* at 2 dpf (M–R) and 5 dpf (S–X) during the assay, with (B, H) basal respiration, (C, I) ATP production, (D, J) maximal respiration, (E, K) proton leak and (F, L) non-mitochondrial respiration. Error bars represent ± SEM calculated from three replicas. The number of animals is indicated below the columns. ^*^P < 0.05, ^**^P < 0.01; unpaired *t*-test.

Mitochondrial oxidative respiration rates were measured in zebrafish larvae to determine the consequences of *wfs1* mutations on mitochondrial function at two developmental ages (2 and 5 dpf) (Fig. 9). The oxygen consumption rate (OCR) was measured before and after the addition of inhibitors (the ATP synthase inhibitor oligomycin; the uncoupling agent FCCP; antimycin A and rotenone) (Fig. 9A, G, M and S) to derive several parameters of mitochondrial respiration (as shown in Fig. 9A). At 2 dpf, *wfs1a* mutant larvae showed no alteration of basal respiration (Fig. 9B), ATP production (Fig. 9C), maximal respiration (Fig. 9D), proton leak (Fig. 9E) or non-mitochondrial respiration (Fig. 9F). *wfs1b* mutant larvae showed significant decreases in basal respiration (Fig. 9H), ATP production (Fig. 9I) and maximal respiration (Fig. 9J) but showed no change in proton leak (Fig. 9K) and non-mitochondrial respiration (Fig. 9L). This suggested that *wfs1b* mutant larvae showed a marked alteration of mitochondrial respiration.

At 5 dpf, both *wfs1a* and *wfs1b* mutant larvae showed no alteration of basal respiration (Fig. 9N and T), ATP production (Fig. 9O and U), maximal respiration (Fig. 9P and V), proton leak (Fig. 9Q and W) and non-mitochondrial respiration (Fig. 9R), with the exception of a decrease in the non-mitochondrial respiration in *wfs1b^W493X^* larvae (Fig. 9X). This suggested that, at 5 dpf, both mutant lines retained a similar metabolic capacity as WT zebrafish.

## Discussion

The aim of the study was to characterize novel zebrafish models of WS. The zebrafish genome contains two *wfs1* genes, *wfs1a* and *wfs1b*. The *wfs1a* gene contains 10 exons and encodes a protein of 1061 amino acids, whereas the *wfs1b* gene contains 8 exons and encodes a protein of 895 amino acids. The human *WFS1* gene contains 8 exons and encodes a protein of 890 amino acids that has a similarity to zebrafish *wfs1a* of 52.47% and to *wfs1b* of 52.80%. This suggested that *wfs1b* gene structure is closer to human *WFS1* than *wfs1a*. We selected the *wfs1a^C825X^* and *wfs1b^W493X^* mutant lines to analyze the effect of a loss of function of each protein on the development of zebrafish larvae, their visual, auditory and locomotor functions and their impact on mitochondrial activity and the ER stress response. During our work, a complementary study to ours was reported by Cairns and colleagues (28), with the characterization of the same *wfs1b^W493X^* fish but with a different *wfs1a* mutant line: *wfs1a^W692X^* (sa10021). While the time points as well as the different *wfs1a* model studied make the direct comparison difficult, their results and ours provide an overall picture of *wfs1* roles in zebrafish during development and adulthood.

Our study shows that the expression patterns of *wfs1a* and *wfs1b* genes are quite distinct and dynamic during early development, which may reflect different functions for the two zebrafish paralogs. Particularly striking is the muscle-specific expression of *wfs1a*, whereas the expression of *wfs1b* is more ubiquitous (Fig. 2, Supplementary Material, Fig. S2)*.* This difference lasts in time as, by 4 months old, *wfs1a* is still highly expressed in muscle compared with *wfs1b* (28). Expression of *Wfs1* in the mouse is also seen in a multitude of different tissues, including skeletal and cardiac muscle (29,30) (see also http://www.informatics.jax.org/gxd/marker/MGI:1328355). *Wfs1*-deficient mice show alterations in protein expression (31) and energy metabolism (32) in both muscle tissue types. *In vitro* assays have also demonstrated that the mammalian Wfs1 protein functions in the nuclear envelope to reposition muscle-specific genes during myogenesis (33). The respiratory failure or dysphagia in WS patients, however, is thought to involve a neurodegenerative rather than muscular pathology, as reviewed in Pallotta *et al*. (34). Dominant missense mutations in human *WFS1* gene are also associated with a distinct Wolfram-like syndrome that includes hypotonia (35). Although there are no overt morphological muscle phenotypes in the single gene mutant zebrafish analyzed here, a metabolic defect in muscle could contribute to the locomotor deficits observed. It is also possible that the mild zebrafish phenotypes reflected transcriptional adaptation triggered by mutant mRNA decay (36), although we did not find evidence for any compensatory up-regulation of paralogous transcript levels by qPCR in the mutants.

Expression of the *wfs1b* gene in the zebrafish pancreas and of *wfs1a* and *wfs1b* genes in the eye and inner ear also appeared to be conserved with that of *Wfs1* in the mammal. Expression of zebrafish *wfs1b gene* was present in the exocrine pancreas from 48 hpf to, at least, 10 dpf. More detailed sections and colocalization studies would be needed to determine expression in the endocrine pancreas and β cells. Apart from the early ubiquitous expression of *wfs1b*, neither gene showed strong expression in the ear or eye at early larval stages (3–4 dpf). However, both genes were expressed in the retina at 7 and 10 dpf (the last stage analyzed) and throughout the otic vesicle. In the retina, the expression of *wfs1b* appeared ubiquitous, whereas levels of *wfs1a* expression differed between the different cell layers. In adulthood, *wfs1b* expression is higher in the eye than *wfs1a* expression. However, these results of reverse transcriptase-PCR do not provide details of the specific sub-localization of each gene expression (28). In the mouse, expression of *Wfs1* mRNA and protein has been reported in all neuronal cell types of the retina (37) and in many cell types of the inner ear (38), including the cochlea and organ of Corti, which have no direct counterpart in the zebrafish ear. Both genes were expressed in the brain at the stages we analyzed (24 hpf–10 dpf), with expression most evident for *wfs1b* at the earlier stages. Cairns *et al*. have reported that expression of *wfs1b* in the eye and brain is stronger than that of *wfs1a* in 4-month-old fish using qPCR (28).

WS is an emblematic disease characterized by optic atrophy, diabetes and neurosensory hearing loss (1). The *wfs1a* loss of function had no effect on the gross morphology of the zebrafish larva except for the size of the ear area that was increased compared with control. No difference in auditory function was observed using ASR, suggesting that the structural anomaly was not correlated to a functional one. Interestingly, a decrease in the size of the rod outer segments was observed in the retina, suggesting that rods degenerated or that the outer segment was shortened. Although these findings are outside the scope of most cases of WS, they are still pertinent to the human pathology. WFS1 expression was faintly detected in human photoreceptors as well as in other species, such as the Cynomolgus monkey (39) and mouse (37,40). In addition, some electroretinogram abnormalities have been reported in human, suggesting some deficit at the photoreceptor level (41). In *wfs1a* larvae, the morphological alteration correlated with a visual deficit, estimated by VMR.

Similar to what we observed with *wfs1a* larvae, *wfs1b* loss of function did not induce any gross morphological alteration. These results are in contrast with those reported by Cairns *et al*., who described shorter larvae for both lines in the early stages of the larvae (30, 50 and 80 hpf). While some alterations, like the head-trunk angle, were fully recovered by 80 hpf, it is not stated whether this difference is the consequence of a developmental delay or a permanent characteristic of the fish (28). Our study would suggest that the developmental defect is transient and limited to the early stages as, by 5 dpf (120 hpf), no morphological differences were noted.

Like *wfs1a* mutant larvae, *wfs1b* mutants have no auditory deficits when analyzed by ASR compared with control. No difference in the number of rods, cones or Retinal Ganglion Cells (RGCs) was observed. Nevertheless, their visual function was altered when measured by VMR and OKR. Indeed, *wfs1a^C825X^* and *wfs1b^W493X^* zebrafish mutants showed a decrease in their distance swum in the dark phase and, for *wfs1b^W493X^* larvae only, in their number of saccades, suggesting a deficit in their visual acuity. In addition, the absence of difference between the mutant lines during the touch escape assay suggested that this alteration is not linked to pure locomotor activity but rather to a pure retinal and/or to a retinal and retinotectal neural network impairment. Indeed, the retinotectal neural network connects RGCs to the optic tectum, a zebrafish brain structure that creates a visual map retinotopically (42).

Cairns and colleagues reported that both *wfs1a* and *wfs1b* mutant fish, at 4 and 12 months old, exhibited a decrease in the RGC density, which was associated with thinner RGC layers and ganglion cell layer (GCL). In addition, in the case of *wfs1b* fish, these morphological observations were correlated to a significantly decreased visual function, recorded by OKR (28). However, we cannot exclude that the absence of *wfs1a* function will not have an impact on the visual function as the size of the rod outer segments was decreased at 5 dpf as well as the RGC density was decreased at 4 and 12 months old. Taken together, these data suggest that the *wfs1b* line might undergo a more severe degenerative process leading to morphological and functional alteration earlier than *wfs1a* fish.

The absence of any gross morphological defect in our fish is also in favor of a retinal and/or retinotectal impairment rather than a muscular or locomotor anomaly. Like Cairns *et al*. (28), we observed a transient developmental defect of the length of the motor neurons of the tail in the 24 hpf larvae, which was completely restored as early as 48 hpf. However, contrary to their study where they reported a decrease in the motor neuron length of the *wfs1b* line (28), we observed an increase in the length of both mutant zebrafish larvae tail motor neurons. This discrepancy between our two studies might be explained by a difference in husbandry conditions (number of larvae/tank and feeding) or the region of the trunk that was analyzed in our study compared with Cairns. For *wfs1a^C825X^*, another possible explanation would be that since the mutation is not the same, it may induce a different phenotype. Nevertheless, since this defect is transitory as evidenced by the normal length of the motor neurons at 48 hpf, more work needs to be done in order to decipher the exact mechanism leading to this early and transient alteration.

Deafness is another hallmark of WS. In humans, hearing loss appears in 46% of the patients (43) in the second decade of life. In addition, in a recent study on a rat model of WS, null for WFS1, hearing loss was observed starting at 6.5 months old, in the higher frequencies, and worsened with age (44). Despite being a prominent feature of WS, none of the characterization studies of the several mouse models of the pathology report any hearing deficit, suggesting either a mild or non-existent hearing loss, or a late onset of the phenotype. In the case of the zebrafish models, it is difficult to determine if the absence of hearing loss is linked to the age of the larvae (5 dpf) compared with humans or if the structure/function of the zebrafish inner ear is too different to that of humans. A longitudinal study, with not only broadband but also frequency-specific stimuli, would help characterize more precisely the impact of the absence of each *wfs1* gene copy on the auditory pathway.

WFS1 protein is involved in the regulation of the UPR. Indeed, it has been shown that WFS1 regulates the ubiquitin ligase HRD1 in order to control the protein level of ATF6. When WFS1 is deficient, HRD1 is less able to address ATF6 to the proteasome (11), leading to an increased activation of ATF6 and to an uncontrolled UPR. In our study, we observed that in basal condition, *wfs1a* gene loss of function impacted to some extent the expression level of *bip*, *atf4α* and *chop*, suggesting that the PERK pathway was altered. In contrast, in the same basal condition, *wfs1b* loss of function impacted the relative expression level of *bip*, *atf4α*, *ire1* and *xbp1us*, suggesting that both the IRE1 and PERK pathways were altered. In stress condition, *wfs1a* gene loss of function marginally impacted the induction of only *perk* and *atf4β*, leading to a preserved UPR. The impact of ER stress was more marked in *wfs1b* mutants since the relative expression level of *bip*, *ire1*, *xbp1s*, *xbp1us*, *perk*, *atf4α* and *chop* was significantly or tended to be altered, confirming that both PERK and IRE1 pathways were affected.

In addition to its role in ER stress, WFS1 is involved in the regulation of the mitochondrial activity in human fibroblasts (9). Therefore, we took advantage of the capacity of the seahorse XFe24 to measure the OCR in the living zebrafish (45) and to decipher the impact of the loss of function of *wfs1a* and *wfs1b* genes on the different mitochondrial parameters. At 2 dpf, *wfs1b^W493X^* mutant larvae showed a decrease in basal, ATP-linked and maximal respiration. *wfs1a^C825X^* did not exhibit any respiration alteration compared with controls. Intriguingly, at 5 dpf, the only observable difference was a decrease in the non-mitochondrial respiration in *wfs1b^W493X^* mutants. These data were in agreement with the decrease in complex I and complex II activities previously reported in patients’ fibroblasts (9) but only in very early developmental stages. In addition, based on our *in situ* hybridization experiments, the expression of *wfs1a* seems to be more tissue-specific than that of *wfs1b*, suggesting a broader impact on mitochondrial physiology for the latter. We can hypothesize that the mitochondrial alteration in tissue(s) expressing *wfs1a* is diluted out in the whole organism experiment.

In conclusion, it appeared that *wfs1a* and *wfs1b* genes recapitulated most of the mammalian *WFS1* roles. However, *wfs1b* may likely be closer to mammal *WFS1* than the *wfs1a* ortholog in terms of cellular expression, function and physiopathology. Taken collectively, our data highlighted the potential of *wfs1b* mutant line as a relevant and promising model to identify a novel therapeutic solution.

## Materials and Methods

### Zebrafish lines and husbandry

The present study followed the recommendations of the ARRIVE guidelines (46) and the European Union Directive 2010/63. Both zebrafish (*D. rerio*) *wfs1a^C825X^* (sa11465) and *wfs1b^W493X^* (sa16422) lines were generated by *N*-ethyl-*N*-nitrosourea (ENU)-induced mutagenesis (Sanger Institute Zebrafish Mutation Project) (27) and were acquired from Zebrafish International Resource Center (University of Oregon, Eugene, OR, USA). A cysteine codon at amino acid position 825 has been replaced with a stop codon for the *wfs1a* line and tryptophan codon has been replaced with a stop codon at position 493 for the *wfs1b* line. Numbering is based on the following accession numbers (XM_690160.9; XP_695252.5) and (XM_679418.7; XP_684510.2) for *wfs1a* and *wfs1b*, respectively. Adult zebrafish were bred and maintained under standard conditions in an automated fish tank system (ZebTEC, Tecniplast) at 28°C, pH 7, conductivity around 500 mS and with a 14 h:10 h light:dark cycle. Eggs were obtained by natural spawning and were maintained in E3 medium (5 mm NaCl, 0.17 mm KCl, 0.33 mm CaCl_2_, 0.33 mm MgSO_4_, 0.05% methylene blue) at 28°C. Each experimental procedure was carried out in triplicate using larvae from three different crosses.

### Genotyping

For isolated homozygous *wfs1a* and *wfs1b* mutant larvae, genomic DNA was extracted from tail fins at 2 months of age and the tissue was lysed in 50 mm NaOH at 95°C for 1 h. Fragments of genomic DNA for the *wfs1*a and *wfs1b* genes were amplified by PCR using GoTaq Green Master Mix (Promega, Madison, WI, USA) and the following primer sequences: *wfs1a*, 5′-TGCGACTCTGGACTCGATTTAG-3′ (forward); 5′-CGGATAAACCTCACCGTAGAGA-3′ (reverse); *wfs1b*, 5′-GGCTATGGTTGCAGTCTTCTTC-3′ (forward); 5′-GCTGAACCAGCATTACAGCTGC-3′ (reverse). These primers amplify a 392 bp region encompassing the *wfs1a* mutation site and a 455 bp region encompassing the *wfs1b* mutation site. These fragments were digested with the restriction enzymes *Mwo*I for *wfs1a* DNA (ER1732, Thermo Fisher Scientific) and *Bsr*I for *wfs1b* DNA (ER0881, Thermo Fisher Scientific). The DNA fragments were separated by electrophoresis on a 3% agarose gel and the genotypes were analyzed. The mutations were confirmed by Sanger sequencing.

### *In situ* hybridization

*In situ* hybridization was performed on AB (ZSB-GENO-960809-7) or *mifta^−/−^* (*nacre^w2^,* lacking melanophores) (47) fish. For RNA probe preparation, two fragments from each gene (*wfs1a*, *wfs1b*) were amplified in a nested RT-PCR that incorporated a T7 polymerase site into the antisense primer and T3 polymerase site into the sense primer. Primers were designed that avoided repetitive sequences, using the reference sequences XM_690160.9 and XM_679418.7 for *wfs1a* and *wfs1b*, respectively. Sequences used were as follows: *wfs1a* 5′ fragment outer primers, 944F 5′-TGTCTTCAGAGAGTAAGTTTGAGCA-3′, 1919R 5′-CTTAATGCTGTGAAGAAAATGGCCA-3′; *wfs1a* 5′ fragment inner primers, 975FT3 5′-AATTAACCCTCACTAAAGGAGGAGAGCAGCCATGACCATGTAC-3′, 1919 T7 5′-TAATACGACTCACTATAGCTTAATGCTGTGAAGAAAATGGCCA-3′; *wfs1a* 3′ fragment outer primers, 1585F 5′-ATCATCTCCAATCTCACCATCGATT-3′, 3327R 5′-GGTCTTCTTTACACGTTGATCCTAGCA-3′; *wfs1a* 3′ fragment inner primers, 1895FT3 5′-AATTAACCCTCACTAAAGTGGCCATTTTCTTCACAGCATTAAG-3′, 3327RT7 5′-TAATACGACTCACTATAGGGTCTTCTTTACACGTTGATCCTAGCA-3′; *wfs1b* 5′ fragment outer primers, 39F 5′-GAGGGTTTATTTAGTCAGCTGCATC-3′, 2164R 5′-GGTGGAGTTGTATACTTTCATCCCT-3′; *wfs1b* 5′ fragment inner primers, 178FT3 5′-AATTAACCCTCACTAAAGCTTAAAAATGGACACGTCTCTGCTT-3′, 1980RT7 5′-TAATACGACTCACTATAGGGCACACTGTTACGATCATTTTGGTCA-3′; *wfs1b* 3′ fragment outer primers, 1810F 5′-TACGCTGCTGCAAGAGTCCACTGTA-3′, 4248R 5′-TTAGAAATTTTGGCATCGGTTCACA-3′; *wfs1b* 3′ fragment inner primers 1849FT3 5′-AATTAACCCTCACTAAAGCTCAGTTGTGGGCTACCTACTTTTT-3′, 3123RT7 5′-TAATACGACTCACTATAGAGCACATTAGGAAAGAGCTCCATTA-3′. Antisense probes generated with T7 polymerase were tested for specific expression patterns, and pairs of probes (5′ and 3′ fragments) for each gene were pooled. *In situ* hybridization was performed as previously described (48,49).

For the stronger staining shown in the retina at 7 dpf and ear at 10 dpf in Figure 2, and in the brain in Supplementary Material, Figure S2, an adapted protocol for larvae post-5 dpf (50) was used. Embryos were mounted in glycerol and were imaged on an Olympus BX51 compound microscope using either bright-field or differential interference contrast (DIC) optics using a Micropublisher 6 camera and Ocular software. Thick sections were cut by hand with a scalpel blade.

### VMR assay

The locomotor activity of zebrafish larvae was quantified with the VMR assay using an infrared (IR) tracking system (Zebralab®, Viewpoint, Lissieu, France) as described previously (51). In brief, at 5 dpf, larvae were transferred in a 96-well plate (Whatman, #7701-1651) with 300 μl E3 medium and the locomotor behavior was monitored with an automated videotracking device (Zebrabox®, ViewPoint). The response to light changes was recorded by an IR camera under IR light illumination. The light protocol was as follows: 30 min of acclimatization in the dark (0% light intensity), then two cycles of 10 min duration light ON (100% light intensity) or light OFF (0%) periods. The total duration of the experiment was 70 min. Activity during the experiment was measured in mm/s. The values obtained during OFF periods were subtracted for each larva from their values registered during ON periods to remove inter- and intra-group variability in basic locomotion.

### ASR assay

The locomotor activity of zebrafish larvae was quantified with the ASR assay in the ZebraBox®. Experimental conditions were similar to those used in the VMR assay. The experiment consisted, first, in acclimating larvae during 30 min with no sound (35 dB ambient), followed by a 1 s stimulation with a white sound at 90 dB, repeated for three times with an inter-trial time interval of 5 min. The quantity of movements during the entire experiment was measured for each larva. Baseline activity levels were subtracted from the activity levels during the sound stimulations (2 min before each stimulation) in order to normalize the values.

### OKR assay

At 5 dpf, zebrafish larvae were immersed in groups of four in a Petri dish (35 mm diameter) containing 2.5% methylcellulose (#9004-65-3, Sigma Aldrich, St Louis, MI, USA). Larvae were placed dorsal side up, forming an X to avoid touching and interfering with each other. All measurements were done in the afternoon between 2:00 p.m. and 6:00 p.m. The room temperature was 28°C and the light was OFF. The visual system performance of larval zebrafish was assessed using a videotracking device (VisioBox®, ViewPoint). Forty 6 mm wide black and white strips were projected at 2 rpm for 1 min clockwise and then for 1 min anti-clockwise. Larvae were illuminated with IR from below and responses were tracked using a FL3-U3-32S2M 1/2.8-inch Monochrome camera (FLEA3, Flir) at 25 frames/s. The number of saccades was manually recorded (PHIVisualize software) and the average number of saccades per 2 min quantified.

### Touch response

To measure the touch escape, 5 dpf larvae were transferred to a rail developed with a 3D printer (18 × 0.4 cm) with 200 μl of E3 medium and placed to the extremity of the rail. The tail of larvae was touched with a tip and the distance traveled was measured during 5 s. The same procedure was repeated three times per larvae, each test separated by 1 min to reduce stress. Each larva was assessed individually and the three values per larva were averaged. The light was ON and the temperature was 28°C.

### Immunohistochemistry

Whole larvae were fixed in paraformaldehyde at 4°C for 48 h, cryoprotected in 30% sucrose and mounted in O.C.T.™ medium (Sakura, Tissue-Tek, Alphen aan den Rijn, The Netherlands). Larvae were transversely sectioned in 10 μm thick slices using a cryostat (Leica, Wetzlar, Germany) at −20°C and were mounted on glass slides. Cryosections were blocked with a solution containing 0.1% Triton X-100 in phosphate-buffered saline (PBS) with 5% Horse serum for 30 min at room temperature. They were subsequently incubated at 4°C overnight with the following primary antibodies: mouse anti-Rho4d2 (1:7000; ab98887, Abcam, Cambridge, UK) and mouse anti-Zpr-1 (1:500; ab174435, Abcam). After several washes, sections were incubated with specific secondary antibodies: Cy3-conjugated anti-mouse IgG (1:800; 715-165-150, Jackson ImmunoResearch, West Grove, PA, USA), Cy3-conjugated anti-rabbit IgG (1:1000; 711-166-152, Jackson ImmunoResearch) or Alexa Fluor-488 conjugated anti-mouse IgG (1:1000; 715–545-150, Jackson ImmunoResearch). Nuclei were counterstained with 40,6-diamidino-2 phenylendole (DAPI; 1:5000; Sigma Aldrich). The emitted fluorescence was measured using a confocal microscope (LSM880 Fast Airyscan, Carl Zeiss, Jena, Germany).

### Whole-mount immunohistochemistry

Whole larvae at 24 and 48 hpf were dechorionated and fixed in paraformaldehyde at 4°C for 24 h, washed with PBS, permeabilized for 2 h in 1% Triton X-100 and then blocked with a solution containing 0.1% Triton X-100 in PBS with 10% Horse serum and 1% BSA for 1 h at room temperature. They were subsequently incubated at 4°C overnight with the mouse anti-SV2 (AB_2315387, 1:50; Developmental Studies Hybridoma Bank, Iowa City, IA, USA). After several washes, larvae were incubated with specific secondary antibodies: Cy3-conjugated anti-mouse IgG (1:500; 715-165-150, Jackson ImmunoResearch), and nuclei were counterstained with DAPI (1:5000; Sigma Aldrich). Z-stack images were acquired using a confocal microscope (LSM880 Fast Airyscan, Carl Zeiss).

### Cell counts

Cone cells, immunolabeled with Zpr-1 antibody, were quantified individually and the total area of rod outer segments, immunolabeled with Rho4d2 antibody, was evaluated and both measures were normalized to the length of the associated retina. Ganglion cells, highlighted by DAPI counterstaining, were counted in three specific regions of the retina, consistent from one sample to another (Supplementary Material, Fig. S2). The total number of ganglion cells from all regions was averaged per larva. The thickness of the GCL was also quantified in the same specific regions of the retina.

### Seahorse XF Mito stress test

The OCR of 5 dpf larvae was measured with the Seahorse XFe24 Extracellular Flux Analyzer (Agilent). Larvae were placed singly in wells of a Seahorse XFe24 spheroid microplate containing 500 μl of E3 medium. A grid was placed manually on larvae to keep them at the bottom of the wells throughout the experiment. The blanks were two empty wells of the plate. Four basal cycle readings were recorded, then five recording cycles following oligomycin (25 μm) injection, five recording cycles after carbonyl cyanide-p-trifluoromethoxyphenylhydrazone (FCCP) (8 μm) injection and nine recording cycles after rotenone + antimycin A (1.5 μm) injection. Calculations for specific parameters, such as non-mitochondrial respiration, basal respiration, maximal respiration, proton leak and ATP production, were made. The room temperature was controlled at 28°C. Measurements of total zebrafish OCR were started immediately and performed according to the manufacturer’s instructions.

### Chemical treatment

To induce ER stress, larvae were incubated, at 4 dpf, for 24 h with 2 μg/ml of tunicamycin (sc-3606, Santa Cruz Biotechnology, Dallas, TX, USA) diluted in 0.1% dimethylsulfoxide (DMSO) directly in the E3 medium. Control larvae were treated with 0.1% DMSO diluted in E3 medium.

### RT-PCR and qPCR

At 5 dpf, total RNA from 20 whole *wfs1a^C825X^*, *wfs1b^W493X^* homozygous mutant fish and its associated phenotypically wild-type controls were extracted using a Nucleospin® RNA Kit (Macherey-Nagel, Hoerdt, France) according to the manufacturer’s instructions. RNA concentration and purity were evaluated using the Agilent RNA 6000 Nano® Kit (Agilent Technologies, Santa Clara, CA, USA). RNA samples (1 μg/μl) were denatured 5 min at 70°C and reverse transcribed into cDNA for 1 h at 37°C using M-MLV reverse transcriptase (Promega). Primer sequences are indicated in Supplementary Material, Table S1. Control reactions were conducted with sterile water to determine the signal background and DNA contamination. The standard curve of each gene was confirmed to be in a linear range, while *zef1α* gene was selected as a reference.

### Western blot

To measure protein expression, 20 whole larvae per condition, at 5 dpf, were homogenized on ice for 15 s in 100 μl of lysis buffer [62.5 mm Tris–HCl, 25% glycerol, 2% sodium dodecyl sulfate, 0.04% cOmplete™ (Roche, Basel, Switzerland), 0.1% PhosSTOP™ (Roche) pH 6.8]. Total proteins were separated on a 1.5 mm 12% running gel and 4% acrylamide stacking gel at 100 V. Proteins were transferred onto a nitrocellulose membrane at 100 V for 1 h in transfer buffer and were blocked in 5% non-fat milk solution for 1 h. Immunoblotting was performed with primary antibodies as follows: rabbit anti-Sigmar1 antibody (1:500, 15168-1-AP; Proteintech, Rosemont, IL, USA), rabbit anti-GADD153 antibody (1:1000, G6916, Sigma Aldrich), rabbit anti-Bip antibody (1:700, SPC-180, Biosciences), rabbit anti-Eif2α antibody (D9G8) (1:500, #3398, Cell Signaling), rabbit anti-p-Eif2α antibody (D9G8) (1:500, #9722, Cell Signaling), in buffer (0.1% TBS/Triton X-100) pH 7.4, overnight at 4°C. After several washes, membranes were incubated with horseradish peroxidase (HRP) conjugated goat anti-rabbit secondary antibody (1:2000; ab6721, Abcam) or goat anti-mouse (1:2000; ab6789, Abcam) secondary antibody for 1 h at room temperature, and the proteins were detected with the indicated HRP detection reagent (10776189, Merck, Germany) and the Bio-Rad imaging system (Bio-Rad, CA, USA). Relative intensities of each band were quantified using Image lab v6.1 software (Bio-Rad) and were normalized to the total protein quantity (Stain-Free™, Bio-Rad) (Supplementary Material, Fig. S4).

### Statistical analyses

Data were expressed as mean ± SEM. Statistical significance between groups was determined by unpaired Student’s *t*-test or two-way ANOVA. The levels of statistical significance considered were ^*^P < 0.05, ^**^P < 0.01 and ^***^P < 0.001. Statistical analyses were performed using Prism v7.0 software (GraphPad, San Diego, CA, USA).

## Supplementary Material

HMG-2021-CE-00815_Crouzier_Supplemental_information_ddac065
