# Supplementary material for: Morphological, behavioral and cellular analyses revealed different phenotypes in Wolfram syndrome wfs1a and wfs1b zebrafish mutant lines
Source: Hum Mol Genet. 2022 Apr 22;31(16):2711–27. doi: 10.1093/hmg/ddac065 (PMC9402244; doi:10.1093/hmg/ddac065)
Supplement: HMG-2021-CE-00815_Crouzier_Supplemental_information_ddac065 [file hmg-2021-ce-00815_crouzier_supplemental_information_ddac065.pdf]

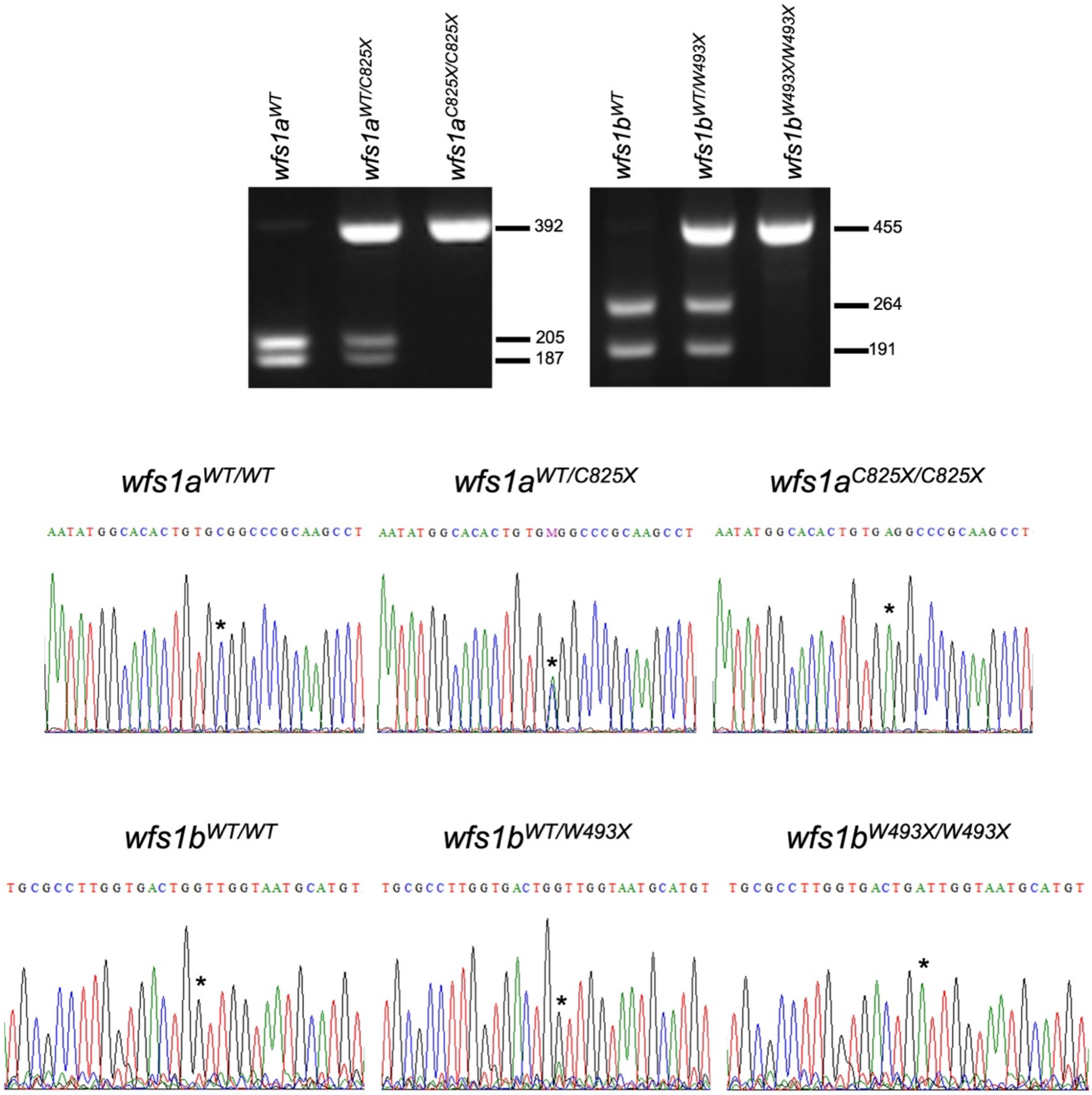

Figure S1. Genotyping procedure for *wfs1a*^{C825X} and *wfs1b*^{W493X}. The wild-type (WT) amplicon for *wfs1a* gave fragments of 205 and 187 bp after *Mwo*I digestion; the WT amplicon for *wfs1b* gave fragments of 264 and 191 bp after *Bsr*I digestion. The *Mwo*I-digested amplicon from homozygous *wfs1a*^{C825X} mutants showed one band at 392 bp and the *Bsr*I-digested amplicon from homozygous *wfs1b*^{W493X} mutants showed one band at 455 bp. Enzymatic digestion of amplicons from heterozygous embryos showed all three bands in each case.
Sanger sequencing of PCR products confirmed the mutation in each zebrafish line. Asterisk (*) denotes the mutated base.

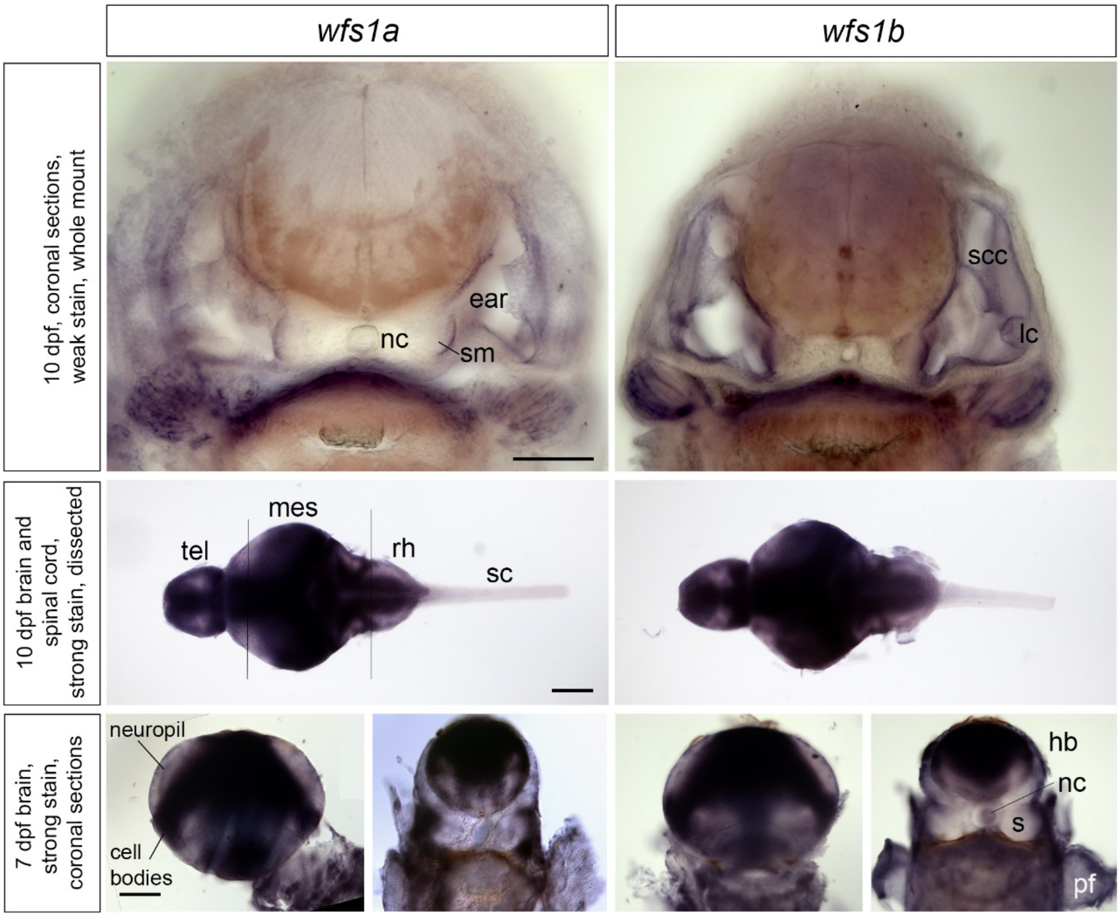

Figure S2. Expression of *wfs1a* and *wfs1b* mRNA in the zebrafish brain at 7 dpf and 10 dpf. In situ hybridizations showing the expression patterns of wfs1a (left-hand column) and wfs1b (right-hand column) at 7 and 10 days post fertilization (dpf). The upper panels show coronal sections through the hindbrain at the level of the ears at 10 dpf. The middle row shows the brain and spinal cord dissected from whole-mount-stained embryos at 10 dpf. Note the absence of stain from the spinal cord. The lower panels show hand-cut sections through the brain at 7 dpf, corresponding to the positions shown by the vertical lines on the 10 dpf brains above. Note the similar patterns between the two genes, with expression marking the cell bodies but not the neuropil of the brain. Abbreviations: hb, hindbrain; lc, lateral crista; mes, mesencephalon; nc, notochord; pf, pectoral fin; rh, rhombencephalon; s, somites; sc, spinal cord; scc, semicircular canal; sm, saccular macula; tel, telencephalon. Scale bars, 100 µm (top and bottom rows); 200 µm (middle row).

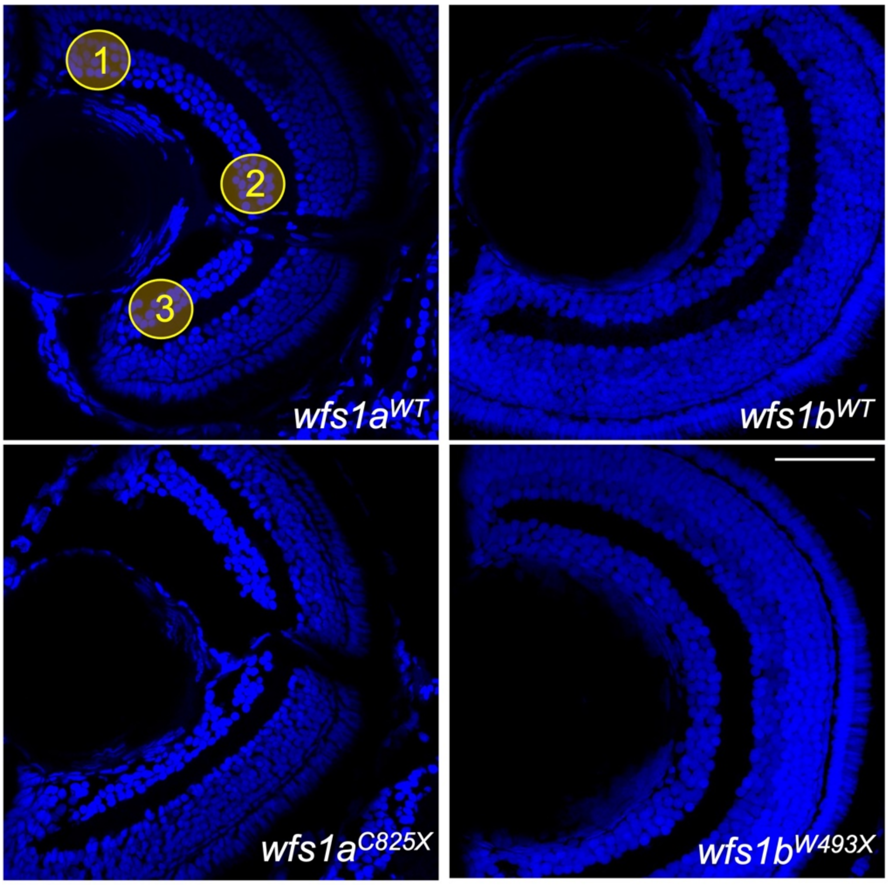

Figure S3. Representative confocal images of the retina of *wfs1a* and *wfs1b* wild-type and mutant larvae at 5dpf, after staining of the retinal cross-sections with DAPI. The numbers indicate the three distinct areas in which the thickness of the ganglion cell layer was measured. Scale bar: 50µm.

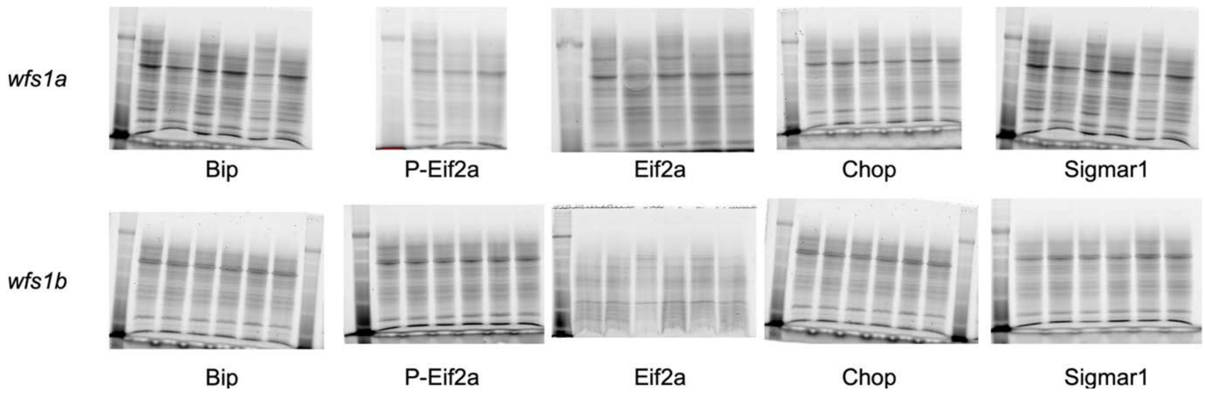

Figure S4. Stain Free for Western blots from Figure 6.

Target genes	Sequences
sigmar1	F 5'-ATGAGCAGGCCTTCTCTAAGGT-3' ; R 5'-TAGTCGTCCCCTCTTTCCATTG-3'
bip	F 5'-AAGAGGCCGAAGAGAAGGAC-3'; R 5'-AGCAGCAGAGCCTCGAAATA-3'
chop	F 5'-CTGATTGGTGCGATGACTGC-3'; R 5'-ACTCGGGCTCCTTCTCTGAA-3'
xbp1us	F 5'-GGGTTGGATACCTTGGAAA-3'; R 5'-AGGGCCAGGGCTGTGAGTA-3'
xbp1s	F 5'-TGTTGCGAGACAAGACGA-3'; R 5'-CCTGCACCTGCTGCGGACT-3'
wfs1a	F 5'-AATGAGGAGCAGTGGGTTGA-3'; R 5'-GGCGAAGGCAAACAAATCGA-3'
wfs1b	F 5'-TCTCTCCAGCCCAATACAGA-3'; R 5'-CATCTGGATGTTTTACTGGC-3'
atf6	F 5'-CTGTGGTGAAACCTCCACCT-3'; R 5'-CATGGTGACCACAGGAGATG-3'
ire1	F 5'-TGACGTGGTGGAAGTTGGTA-3'; R 5'-ACGGATCACATTGGGATGTT-3'
hsp90b1	F 5'-GGCGTTAATCTGCTATTGAG-3'; R 5'-GTCTTTGGTTTGTCCTTGTC-3'
atf4α	F 5'-CCGGGAATCATGGCAGTGTA-3'; R 5'-GAGAAGCTGCGGTATTTGCG-3'
atf4β	F 5'-TGACCCTCTGCGGTCAATTC-3'; R 5'-ACGAATGATCTTCACCACTGTCT-3'
perk	F 5'-TGGGCTCTGAAGAGTTCGAT-3'; R 5'-TGTCAGCCTTCTCCGTCTTT-3'
zef1α	F 5'-TTCTGTTACCTGGCAAAGGG-3'; R 5'-TTCAGTTTGTCCAACACCCA-3'

Table 1. Primers used in this study in Figures 6 and 7.
